# Navigating challenges and adherence in time‐restricted eating: A qualitative study

**DOI:** 10.1111/1747-0080.12922

**Published:** 2025-02-02

**Authors:** Hilmi S. Rathomi, Nahal Mavaddat, Judith M. Katzenellenbogen, Sandra C. Thompson

**Affiliations:** ^1^ School of Population and Global Health University of Western Australia Nedlands Western Australia Australia; ^2^ Faculty of Medicine Universitas Islam Bandung Bandung Indonesia; ^3^ UWA Medical School University of Western Australia Nedlands Western Australia Australia; ^4^ Western Australian Centre for Rural Health University of Western Australia Nedlands Western Australia Australia; ^5^ School of Allied Health University of Western Australia Nedlands Western Australia Australia

**Keywords:** behaviour change, dietary adherence, facilitators and barriers, qualitative study, time‐restricted eating

## Abstract

**Aims:**

Adherence to any dietary approach is crucial for achieving long‐term benefits. This qualitative study aims to explore the facilitators and barriers to adherence, and how individuals in community settings navigate time‐restricted eating in their daily lives.

**Methods:**

Semi‐structured, in‐depth interviews were conducted with 21 participants who had practised time‐restricted eating (confining the daily eating window to <10h a day; and excluding periodic fasting methods like the 5:2 approach or alternate day fasting) for periods ranging from 3 months to more than 5 years. A qualitative content analysis, underpinned by the Capability‐Opportunity‐Motivation‐Behaviour Model, identified multiple facilitators, barriers, and strategies that evolved over the practice.

**Results:**

Key facilitators included the simplicity and versatility of time‐restricted eating, maintaining a non‐obsessive and non‐dieting mindset, and having a supportive environment. Barriers included hunger and food cravings, an obsessive mindset during the initial stages, and conflicting schedules with social eating occasions, including holidays. Participants employed several coping strategies to successfully navigate adherence and reported confidence in maintaining time‐restricted eating as a lifestyle that contributes to better health and weight management.

**Conclusion:**

Our findings suggest that successful implementation of time‐restricted eating in community settings requires flexibility and viewing it as more than a short‐term weight loss tool. Guidelines are needed to help individuals and practitioners implement better practices and promote healthier behaviours.

## 1 INTRODUCTION

Time‐restricted eating (TRE) is a dietary approach that limits the timing of food intake to specific windows each day, such as 8 am to 6 pm or 12 pm to 8 pm, with water‐only fasting during the remaining hours.^1^, ^2^ This approach has gained increasing attention in recent years for its potential health benefits, particularly for weight management. It has shown promise in clinical trials as an effective and feasible dietary intervention for managing weight and improving metabolic health, including reducing blood pressure, improving blood glucose levels, and lowering cholesterol.^3^, ^4^, ^5^ Unlike traditional energy‐restricted diets that focus on reducing overall energy intake, TRE primarily regulates the timing of eating, allowing individuals to consume their daily energy intake within a designated time frame, typically ranging from 4 to 12 h.^1^, ^2^, ^6^ This approach aims to align eating behaviour with circadian rhythms, balancing eating times to meet the body's need for nutrients, and fasting periods that activate autophagy and cell repair.^1^, ^7^ Moreover, confining the eating window leads to extended periods of fasting which promotes fat oxidation and helps reduce excess weight and fat.^8^ Additionally, studies have shown that time‐restricted eaters unintentionally reduce their daily energy intake by 10%–30%, due in part to fewer opportunities to eat later in the evening.^2^, ^9^ As traditional energy‐restricted diets often face challenges with long‐term effectiveness, mainly due to difficulties with adherence,^10^, ^11^ there is a need to explore alternative approaches such as TRE.

Research on the feasibility of TRE in clinical trials has shown it to be a viable approach for weight management. A systematic review by Termanssen et al. found that the average recruitment rate and adherence in trials were high (95% and 89%, respectively), suggesting that volunteers were keen to explore TRE as a dietary alternative.^12^ On the other hand, Oneal et al. summarised barriers to adherence to TRE within clinical trials, primarily related to social events, work schedules, family, and psycho‐physical factors.^3^ Oneal et al.'s study also suggested strategies to improve adherence in clinical trials, emphasising flexible protocols and providing support and education to participants.

Despite the promising findings from trials, little is known about how individuals adhere to TRE in uncontrolled, free‐living environments. These settings often introduce complexities not accounted for in clinical trials. In real‐world contexts, eating events serve not only as a source of physical nourishment but also as social occasions, where individuals are known to frequently adjust their eating times based on work schedules, food availability in the home, and personal preferences.^1^, ^13^ Qualitative approaches are particularly well‐suited to capturing the nuances of real‐world adherence by exploring how individuals perceive, adapt to, and navigate the barriers and facilitators to adopting TRE in their daily lives. While O'Connor et al. examined facilitators and adherence among community‐dwelling individuals, their research was conducted during the COVID‐19 pandemic, when social interactions were significantly restricted.^14^ Therefore, further research is required to address this gap by providing insights from more contemporary times and settings with the resumption of social activities.

Our study aims to explore the facilitators and barriers to TRE adherence among free living individuals, as well as how they navigate the challenges of maintaining TRE. Understanding these aspects among independent‐living individuals is an important step to inform the development of comprehensive guidelines on TRE practice as part of weight management strategies that can benefit patients and practitioners.^15^

## 2 METHODS

This study is part of a larger qualitative research project examining the acceptability and experiences of TRE among free‐living individuals. Related manuscripts have examined complementary topics, such as motivations for adopting TRE and its biopsychosocial impacts, which do not overlap with this study's specific focus on adherence. In this article, we concentrate on understanding the facilitators and barriers influencing individuals' adherence to TRE. We purposively recruited adults (aged ≥18 years) residing in Western Australia who had been utilising TRE every day for at least 3 months to ensure participants had ample time to immerse themselves in the approach. TRE was defined as a daily eating window of <10 h, excluding periodic fasting methods like the 5:2 approach or alternate day fasting. Participants were not excluded based on clinical conditions or their reasons for practising TRE, as our aim was to capture a broad range of experiences regarding TRE. Recruitment methods included social media posts, flyers in public places, university websites, and radio interviews.

Ethics approval for this study was obtained from the University of Western Australia Human Research Ethics Committee (Project Number: 2021/ET000993). Participants were fully informed about the study's purpose, process, and their rights, and written informed consent was obtained from all participants. Confidentiality of participant information was maintained throughout the study.

Data collection occurred from October 2022 to May 2023. Interested individuals received detailed information about the study and provided informed consent before participating in semi‐structured in‐depth interviews. These interviews explored participants' experiences with TRE, covering topics from initial discovery to daily maintenance and long‐term plans. The interview guide was developed with input from experts and a consumer representative, and it was pilot tested with individuals having characteristics similar to targeted participants. The topics covered in the interviews relevant to the study focus are listed in Box 1. As this is part of a larger study, a comprehensive description of the interview guide development process, along with the full set of guiding questions, is available in a related paper,^16^ as well as in Table S1.

BOX 1 **List of interview topics relevant to the study focus.**

*Interview topics*:

1. Description of a typical day when practising TRE, focusing on meal timing
2. Experience with daily TRE
3. Reasons for sticking with TRE
4. Obstacles or challenges experienced with TRE and how to handle them
5. Family and friends' views on practising TRE
6. Future plans to control weight related to TRE

Abbreviation: TRE, time‐restricted eating

The first author, a male GP with qualitative research experience and training, conducted all interviews. Interviews were conducted online using video conferencing or face‐to‐face on university premises and lasted from 30 to 90 min. Audio recordings were transcribed verbatim using Otter.ai software and reviewed manually. Interviews and analysis were conducted concurrently. Sampling concluded when no new data contributed additional insights to the broader research project, which encompassed motivations, adherence, and impacts of TRE. For this specific study, saturation was observed in relation to barriers, facilitators, and adherence‐related themes, ensuring comprehensive coverage of participants' experiences.

We analysed the interview transcripts using conventional content analysis.^17^ Two researchers independently coded the data, engaging in collaborative discussions with co‐researchers to explore and refine emerging themes. Some initial codes were combined during this process. Refined codes were then sorted into categories based on how different codes were related and linked, following a systematic process with the assistance of NVivo software (Release 1.7.1, QSR International Pty Ltd).

To guide the analysis, we employed a hybrid approach. Initial coding was conducted inductively, allowing themes to emerge naturally from the data without the application of a predetermined framework. However, as the analysis progressed, the richness of the data highlighted the need to structure these findings within a theoretical framework for enhanced interpretation. Consequently, the emergent themes were mapped to the Capability‐Opportunity‐Motivation‐Behaviour (COM‐B) model^18^, which provided a comprehensive lens for understanding the barriers and facilitators to TRE adherence.

The COM‐B model was chosen for its comprehensive coverage of behavioural factors, providing a nuanced understanding of those influencing TRE adherence, as well as a solution‐oriented approach applicable to various health‐related behaviours.^18^ This model frames adherence as a function of three interrelated components: capability, opportunity, and motivation. Capability refers to the individual's psychological and physical capacity to engage with TRE, including having the necessary knowledge and skills. Opportunity encompasses all factors outside the individual that make TRE possible, including physical and social opportunities. Motivation involves all brain processes that drive TRE practice, including conscious decision‐making, habitual processes, and emotional responses.^18^

Mapping the findings to the COM‐B framework provided structure to the analysis and extended the focus to potential interventions for improving TRE adherence.^18^, ^19^, ^20^ The COM‐B model has been widely used in dietary and nutrition research to assess adherence, identify facilitators and barriers, and guide intervention design.^20^, ^21^ This hybrid approach reflects a balance between grounding findings in participants' subjective experiences and situating them within a broader theoretical context.

We employed several strategies to ensure rigour and trustworthiness. Member checking involved returning interview transcripts or summaries to participants to ensure their perspectives were accurately represented, provide opportunities for reflection on personal experiences, and allow for additional data contributions.^22^ This process also facilitated shared meaning‐making between researchers and participants. Investigator triangulation was used to compare coding results between two researchers, fostering reflexivity and a nuanced interpretation of the data. Debriefing sessions with other team members allowed for collaborative reflection and deeper exploration of emerging themes. Additionally, we provided detailed descriptions of the research context and setting to support contextual richness and transferability of the findings.^23^

This study is grounded in a constructivist epistemology, which emphasises the subjective experiences and contextual realities of individuals practising TRE. By focusing on participants' unique perspectives and the co‐construction of meaning during the interview process, this approach aligned with our aim to explore the nuanced barriers and facilitators to TRE adherence in free living settings.^24^ The first author, also a TRE user, adopted a reflexive stance throughout the research process, acknowledging his positionality and engaging in collaborative discussions with co‐authors to ensure a balanced interpretation of the findings. This study is reported according to the Consolidated Criteria for Reporting Qualitative Research (COREQ) guidelines^25^ (Table S2).

## 3 RESULTS

Twenty‐one participants were recruited, ranging in age from 27 to 60 years (median 48), with the majority being female (*n* = 15), and all but one living with family. Occupations varied, including full‐time work, self‐employment, and fly‐in‐fly‐out roles, where workers travel to remote work sites for a period and then return home for time off. More than half (*n* = 11) had practised TRE for over 12 months, with some maintaining TRE for more than 5 years. Among those practising TRE for <12 months, 4 were relatively new (<6 months). Eating windows varied, with most adhering to a 6‐ to 8‐hour window, and three participants following the One‐Meal‐A‐Day approach. The most common eating window was from 12 pm to 6 pm (*n* = 10), with only two participants starting before the afternoon. Detailed characteristics of each participant are listed in Table 1.

**TABLE 1:** Participants characteristics.

No	Age	Gender	Occupation status	Eating window	Eating time	Practice duration
1	52	F	Self employed	6–8 h	12 pm to 6 pm	>12 months
2	51	F	Self employed	One meal a day	12 pm to 2 pm	6–12 months
3	58	F	Not working	6–8 h	12 pm to 6 pm	6–12 months
4	43	F	Shift work	8–10 h	10 am to 6 pm	>12 months
5	54	F	Full‐time	6–8 h	12 pm to 8 pm	>12 months
6	40	F	Full‐time	4–6 h	4 pm to 7 pm	>12 months
7	55	F	Part time	4–6 h	12 pm to 6 pm	6–12 months
8	60	F	Shift work	6–8 h	11 am to 7 pm	>12 months
9	40	F	Part time	4–6 h	1 pm to 6 pm	3–6 months
10	48	F	Self employed	One meal a day	3 pm to 5 pm	3–6 months
11	52	M	Full time	One meal a day	5 pm to 6 pm	3–6 months
12	43	F	Full time	6–8 h	1 pm to 8 pm	>12 months
13	29	F	FIFO	4–6 h	12 pm to 7 pm	>12 months (5 years)
14	40	M	Full time	8–10 h	12 pm to 8 pm	>12 months (5 years)
15	43	F	Full time	8–10 h	9 am to 5 pm	6–12 months
16	60	F	Retired	4–6 h	1 pm to 6 pm	>12 months
17	52	M	Full time	6–8 h	12 pm to 6 pm	6–12 months
18	27	M	University Student	8–10 h	1 pm to 9 pm	>12 months (5 years)
19	48	M	Full time	4–6 h	12 pm to 6 pm	3–6 months
20	49	M	Full time	6–8 h	12 pm to 6 pm	>12 months
21	27	F	Full time	6–8 h	12 pm to 6 pm	>12 months (5 years)

Abbreviations: F, female; FIFO, fly‐in‐fly‐out; M, male.

Participants reported that the facilitators and barriers to regular TRE practice shifted dynamically over time, with different factors influencing adherence at various stages, such as during the initial adoption phase and after several months. A summary of facilitators and barriers mapped according to the components of the COM‐B Model is shown in Table 2.

**TABLE 2:** Barriers and facilitators of time‐restricted eating practice mapped to Capability‐Opportunity‐Motivation‐Behaviour model components.

COM‐B model component	COM‐B sub‐component	Facilitators	Barriers
Capability	Physical capability	Progressive ease of TRE Practicality of TRE concept Physically adapted to fasting	Hunger and food (sugar) craving
	Psychological capability	Simplicity of TRE Versatility in adjusting eating times Adequate comprehension of the TRE mechanism	Temporary obsession with watching the clock
Opportunity	Physical opportunity	Low cost to adhere to TRE Alignment with daily schedule No strict restriction of doing TRE	
	Social opportunity	Cultural compatibility Independent living situation Support from family and companions Respectful environment	External disapproval Conflicting schedule with social eating occasion including holiday
Motivation	Automatic motivation	Rapid weight loss Experiencing positive health impact	
	Reflective motivation	Feeling of control Getting positive feedback from others Non‐dieting mindset Non‐obsessive mindset	Obsessive and binge eating mindset Preoccupation with weight loss

Abbreviations: COM‐B Model, Capability‐Opportunity‐Motivation‐Behaviour Model; TRE, time‐restricted eating.

Experiences with daily TRE varied among participants but generally included an initial adjustment period followed by a sense of routine and ease for most participants. Initially, many reported struggling with hunger and the challenge of resisting certain mealtimes and eating at social events. Obsession with watching the clock to eat exactly at the start of their eating windows was common at the beginning. However, over time—typically between 3 and 8 weeks—many found that their bodies adapted, and their hunger reduced. They also noticed weight reduction, improvements in their energy levels, and mental clarity during fasting periods. Some participants enjoyed the simplicity TRE brought to their daily schedules, as they no longer needed to plan multiple meals throughout the day.

And the first few weeks were hard, we'd be getting to 10 o'clock and watching the clock. By the time 11 o'clock came, it was … just give me more. We gradually pushed it out, we'd go to 11.30 to 12 o'clock. And now we open our eating window about 12.30. Sometimes it's 1 pm (P16, F, 60, eating window from 1 to 6 pm).

I was shocked in that first three‐month period, that I needed to be quite strict, and I needed to have some rules in place for myself. And then as I found that I was losing the weight, it was easier to stay motivated, I guess. And but now I'm a bit more flexible with the times and things like that (P5, F, 54, eating window from 12 to 8 pm).

Participants described several factors that made it easier for them to adhere to TRE. These factors included those which kept or enhanced their motivation, made them feel capable of practising TRE, and allowed them to continue with TRE. These included factors related to the nature of TRE features, such as its simplicity, practicality, low cost, lack of strict restrictions or prescriptions regarding what or how much to eat, as well as the logical scientific concepts behind TRE practice. Participants valued the flexibility to choose their eating window based on their preferences, finding adaptability helpful in sticking to TRE while managing their personal circumstances.

If there's a family event like a family celebration, either you say, ‘Sorry I'm not eating at this time’ or I'm going to eat outside my eating window and then the next day you just get back onto your fasting window again. So, to me it's really versatile and it doesn't cost anything (P16, F, 60, eating window from 1 to 6 pm).

Other enablers were related to individual conditions, including physical adaptation to fasting, experiencing less hunger over time, and becoming accustomed to the routine. Factors such as cultural compatibility, independent living situations, an adequate understanding of TRE's physiological basis, and viewing TRE not as a diet but as a long‐term lifestyle also contributed to success with the method.

It was just very logical that this is the correct way to do, to have lesser meals. And TRE is actually healthier for you. It was actually very easy and doesn't feel like I'm putting in any effort (P18, M, 27, 1–9 pm).

A positive attitude from family, friends, health professionals, and supportive communities served as motivation for many participants. While some received strong encouragement, particularly from family members who also practised TRE, others encountered scepticism or lack of support, especially when social mealtimes conflicted with their TRE schedule. Additionally, having family members with different eating schedules or needing to prepare shared meals posed further challenges. Regardless of the reactions, participants consistently highlighted the importance of having a respectful and non‐judgemental environment to practise TRE.

I think it really helps having my husband do it at the same time. Because I'm sure that if I saw him eating or doing other things, like if we were sitting on the lounge at night time, and he was snacking, I would want a snack too. So, the fact that he's not doing that really helps (P9, F, 40, eating window from 1 to 6 pm).

So, I don't really have to worry about support because I can just do it myself. I don't know if support itself is important, I can keep myself motivated. I just don't want other people to distract me. It's like anti support is more of a problem than lack of support (P15, F, 43, eating window 9 am to 5 pm).

Another key facilitator was the health benefit that participants experienced. Many reported weight loss, improved digestion, fewer health issues, enhanced mental clarity, and increased energy levels as reinforcing factors for maintaining TRE.

I think it took about six or eight weeks before I noticed a reasonable loss of weight. And then, it just continued, and it was enough to keep me motivated to keep going (P5, F, 54, eating window from 12 to 8 pm).

Participants faced obstacles such as hunger, food cravings, conflicting schedules, social constraints, and an obsessive mindset. Hunger and cravings were particularly challenging during the initial adjustment period.

If we close our window with a sugar type dessert, then we have a really strong craving to eat through the night (P11, M, 52, eating window 5 to 6 pm).

Obviously, the first few weeks—maybe three or four—weren't exactly difficult, but I did find myself constantly watching the clock, waiting until I could have lunch. But, once I got used to it after those first weeks, it became easier and easier (P5, F, 54, eating window from 12 to 8 pm).

Conflicting schedules were another significant barrier, as the designated eating window sometimes clashed with personal or professional commitments. This issue was exacerbated during holidays when more social eating was expected.

I think any difficulties that I really have sometimes, which makes it a bit challenging, if I want to go and have a coffee with a friend, sometimes they'll order some cake or something like that. But I just order a black coffee and just say, I'm not really all that hungry (P1, F, 52, eating window from 12 to 6 pm).

One participant mentioned that an obsessive mindset or preoccupation with weight had previously led to failure in practising TRE. After managing this mindset, she realised that unrealistic expectations of rapid weight loss were a barrier to maintaining TRE.

Once I knew that I could not to expect weight loss straightaway and I knew all those kinds of things [TRE mechanisms], I knew these were the reason why I gave up the first time when I did it with no knowledge. It was game changer (P10, F, 48, eating window from 3 to 5 pm).

Participants used several approaches to navigate adherence to TRE and plan for long‐term practice. Table 3 summarises these strategies for coping with various barriers.

**TABLE 3:** Approaches used for navigating challenges in time‐restricted eating practice.

Coping approaches	Barriers				
	Food cravings and Hunger	Conflicting Schedule	External pushback	Obsessive mindset	Preoccupation to weight
Having flexible window and positive mindset	X	X	X	X	
Finding helpful resources			X	X	X
Coping with hunger (drink plenty of water, keep busy and find distraction)	X				
Making dietary adjustment (reduce sugar and carbs)	X				
Vigilance without obsession		X	X	X	X

A flexible eating window and positive mindset were critical for managing food cravings, hunger, and scheduling conflicts. Flexibility allowed participants to adapt TRE to their lifestyle more easily, reducing stress and increasing adherence. Helpful resources and supportive communities also played a role in maintaining TRE amidst family routines. To manage hunger, many practised consuming coffee, water, or non‐caloric beverages and staying busy to distract from thoughts of food. Adjusting food selections to prioritise nutrient‐dense options and reduce sugar also helped.

I've discovered that when I get hungry, I know it's temporary. And if I keep myself busy or just wait a bit longer, that hunger will pass. And then I can keep fasting. It helps to have that knowledge to know what's going on in your body (P7, F, 55, eating window from 12 to 6 pm).

So, eating the vegetables and the protein, increasing the protein, that's making me feel fuller for longer (P16, F, 60, eating window from 1 to 6 pm).

More experienced TRE users practised vigilance to maintain a shorter eating window and healthier food options without obsessing over their weight or strict timing. This balanced approach helped them navigate challenges more effectively and sustain their TRE practice long‐term.

If we have a social event, we just enjoy it. If it was an evening event, we go out, enjoy food or drinks, whenever we're there. And we just, carry on as normal the next day (P9, F, 40, eating window from 1 to 6 pm).

All participants in our study expressed a desire to continue practising TRE long‐term as part of their weight management and healthy lifestyle strategy. They were confident in their ability to sustain TRE and integrate other healthy habits, hoping for more positive views from health practitioner and wider communities.

I'm very confident that I will be able to maintain this [TRE] and make this a lifelong journey. And I don't want to go back to eating all the time and how I was feeling all the time. And I'm confident that I will keep doing it (P4, F, 43, eating window 10 am to 6 pm).

Hopefully I see more of it coming from the doctors, not doctors being afraid of it. … the more the podcasts get out there and the Facebook groups support people. And older people with a lot of health conditions are doing it and winning. And it's a lifestyle, not a diet. There's always a fresh start tomorrow. And the support in your ears is so important (P3, F, 58, eating window from 12 to 6 pm).

## 4 DISCUSSION

This study uniquely contributes to the TRE literature by examining real‐world adherence among free‐living individuals, a context often overlooked in prior controlled trials. Unlike previous research limited to trials or clinical settings, our findings provide insights into how TRE is navigated in everyday life, emphasising the importance of flexibility and social support. By mapping facilitators and barriers using the COM‐B model,^18^ this study offers a structured understanding of the behavioural factors influencing TRE adherence. Specifically, we found that physical and psychological capability were key facilitators, whereas social opportunity presented the most significant barrier.

All participants expressed a long‐term commitment to TRE, aiming to sustain it as a lifestyle rather than a short‐term dietary measure. This commitment was attributed to the flexible and non‐dieting approach many adopted, aligning with findings from prior studies on sustainable weight management.^14^, ^26^, ^27^, ^28^, ^29^ Participants described TRE as a simple, straightforward, manageable and cost‐free method to integrate healthy eating into their lives. This was contrasted with other dietary approaches, such as calorie‐restricted, Mediterranean, or other specialised diets that often require extensive planning and external support and are associated with high costs.^30^, ^31^, ^32^, ^33^ However, the costs associated with these diets may vary across demographics and locations.^34^, ^35^ These factors likely contributed to high adherence observed among our participants.

Positive outcomes, such as weight loss, improved mood, increased energy, less pain, and fewer health complaints, reinforced adherence to TRE. While experiencing positive impacts is a common facilitator for any weight control method,^21^, ^36^ the sense of control, flexibility, and the absence of a dieting mindset appear to set TRE apart from other approaches. Social support also played a crucial role, aligning with previous studies emphasising the importance of a supportive environment during the early stages of TRE.^14^, ^37^ However, participants mostly valued a non‐judgmental and respectful environment, enabling them to practise TRE independently. This aligns with findings from Fanaroff et al. where external intervention did not significantly improve adherence to TRE, highlighting that TRE adherence depended more on internal motivation.^38^

The primary barriers to TRE practice, including hunger and preoccupation with meal timing, were typically transient and decreased within 3–8 weeks as participants adapted. Although barriers we found were similar to those reported in earlier studies,^14^, ^29^, ^39^ our participants reported fewer overall barriers. This may be due to our focus on long‐term TRE users, who may have overlooked early‐phase challenges.^40^ The low barriers perceived might also be attributed to participants adopting strategies such as reducing sugary foods, increasing protein intake, drinking plenty of water, and staying busy, to mitigate these challenges. The few barriers reported in our study contrast with other dietary approaches, where more barriers than facilitators were generally identified, including complex behaviour changes and strict restriction.^21^ Some studies suggested that gradually increasing fasting duration and starting with a stricter initial period of implementing TRE, followed by more flexibility tailored to individual conditions, can improve adherence,^29^, ^37^ as seen among participants in our study.

One persistent barrier was the challenge of maintaining TRE during social occasions, which is consistent with previous studies in both RCTs and community settings.^3^, ^14^, ^28^, ^39^, ^41^ However, barriers related to social eating occasions are not exclusive to TRE, but also occur with other approaches, such as with low‐calorie diets, where users often need to avoid social engagements or unavoidably deviate from their programme.^33^, ^36^

Some studies reported that social evening activities was the largest barrier for many TRE users.^39^, ^42^ However, many participants addressed this by extending their eating windows on weekends or choosing later eating windows to accommodate social meals, such as dinners. These adjustments allowed participants to balance adherence with social engagement, though early TRE has been associated with greater metabolic benefits, including weight loss, glycaemic control and blood pressure.^43^ Choosing an eating window between early or late TRE should be an informed decision that allows individuals to balance optimising health benefits and sustainability. This underscores the importance of personalised advice from health practitioners, enabling individuals to select an eating window that suits their circumstances.^39^

Our inductive data analysis allowed themes to emerge organically rather than being shaped by a predefined model, yet the facilitators and barriers to TRE adherence aligned well with the components of the COM‐B model.^18^ In terms of capability, we observed that physical ease and practical adaptability were facilitators, as participants adjusted to fasting over time, although some initial challenges with hunger and clock‐watching were noted. Opportunity factors included the low cost and compatibility of TRE with daily schedules, supporting participants' adherence, though social barriers, such as conflicting schedules and external disapproval from family and friends, posed challenges, particularly around social occasions. Motivation factors such as the health benefits and non‐dieting mindset were instrumental in sustaining TRE, despite some participants experiencing obsessive thoughts about weight loss in early stage. These findings, mapped to the COM‐B model, underscore how physical, social, and motivational factors collectively shape TRE adherence, highlighting areas where targeted support could enhance long‐term success.

Our findings underscore the importance of interventions tailored to the COM‐B components, particularly enhancing social opportunity. Practical strategies, such as flexible eating windows and a positive mindset, may improve adherence by enhancing capability, motivation, and social opportunity. Although TRE shows promise as a sustainable dietary approach, further research is needed to investigate its long‐term implications, including sustained adherence and applicability, as its suitability for various populations and practitioners has not yet been fully established.^44^

Our findings suggest that interventions to enhance social support and address psychological barriers, especially during early stages, could improve long‐term adherence. As interest in TRE grows, incorporating it into dietary guidelines could provide clearer, evidence‐based advice for practitioners and the public, rather than relying on less trusted sources. Future research should explore the challenges faced by individuals who have not successfully adopted TRE, as well as examine strategies to address the social and environmental barriers identified in this study to adhering to TRE. Such research could provide actionable insights to support practitioners in tailoring their recommendations and enhancing patient readiness for TRE practice.

This study contributes to the limited understanding of real‐life TRE practice in community settings, addressing gaps in previous research conducted during the COVID‐19 era.^14^ By including a diverse range of participants across various ages, genders, occupations, and backgrounds, our findings capture a broad spectrum of personal experiences with TRE. The use of the COM‐B Model in this study structured the interpretation, offering a nuanced understanding of the factors to TRE adherence, to inform further interventions needed to improve TRE adherence, and enabled comparison with other dietary approaches.^18^, ^20^, ^21^, ^45^

However, there are some limitations to our study. As this study draws on self‐reported experiences, the findings reflect participants' subjective interpretations and may be shaped by their perspectives or a desire to present their experiences positively. Our recruitment through social media platforms may have attracted individuals already inclined toward TRE, which could influence the variety of perspectives and experiences included. By focusing on regular TRE users who had been practising for over 3 months, our findings do not fully capture the initial challenges faced by new adopters or those who discontinued. The sample also had limited gender diversity and lacked representation from rural populations and broader cultural backgrounds. Furthermore, as we recruited adults under 60 years in Western Australia, the findings may not be directly transferable to other regions or age groups. These limitations may affect the transferability of the findings to other demographic or cultural contexts. Future research should aim to include more diverse populations to provide a broader understanding of TRE experiences.

In conclusion, this study highlights the importance of flexibility and viewing TRE as more than a short‐term weight loss tool. Clinicians should personalise TRE advice to individual circumstances. Given the participants' commitment to long‐term TRE, guidelines are necessary for optimal practice. Since social barriers present significant challenges, future research should focus on improving social opportunities by fostering a respectful, non‐judgemental environment and encouraging family support, to enhance adherence and optimise health outcomes. Furthermore, a study exploring social aspects in greater depth could provide valuable insight into the dynamics of social barriers and facilitators to TRE adherence.

## AUTHOR CONTRIBUTIONS

Hilmi S. Rathomi conceptualised and designed the study, developed interview guide, conducted the literature search, interviewed participants, manually verified transcript accuracy, anonymised data, performed coding and analysis, drafted the manuscript, and conducted critical revisions. Nahal Mavaddat contributed to the concept and design, served as the second coder, participated in analysis, provided input on the interview guide, critically revised the manuscript, and gave final approval. Judith M. Katzenellenbogen contributed to the concept and design, provided input on the interview guide, critically revised the manuscript, and gave final approval. Sandra C. Thompson contributed to the concept and design, provided input on the interview guide, critically revised the manuscript, and gave final approval. All authors have reviewed and approved the final manuscript and confirm that the content has not been published elsewhere.

## FUNDING INFORMATION

HSR received financial support from the Indonesia Endowment Fund for Education (LPDP) for a doctoral degree.

## CONFLICT OF INTEREST STATEMENT

The authors declare that there are no conflicts of interest.

## ETHICS STATEMENT

This study was approved by the University of Western Australia Human Research Ethics Committee (project number 2021/ET000993). Participants were fully informed about the study's purpose, process, and their rights, and written informed consent was obtained from all participants. Confidentiality of participant information was maintained throughout the study.

## Supporting information


**Table S1.** Complete interview guiding questions.
**Table S2.** COREQ Checklist for manuscript ‘Navigating Challenges and Adherence in Time‐Restricted Eating: A Qualitative Study’.

## Data Availability

The data that support the findings of this study are available on request from the corresponding author. The data are not publicly available due to privacy or ethical restrictions.
