# Supplementary material for: Navigating challenges and adherence in time‐restricted eating: A qualitative study
Source: Nutr Diet. 2025 Feb 2;82(4):423–33. doi: 10.1111/1747-0080.12922 (PMC12401815; doi:10.1111/1747-0080.12922)
Supplement: Supplementary file 1 — Table S1. Complete interview guiding questions. Table S2. COREQ Checklist for manuscript ‘Navigating Challenges and Adherence in Time‐Restricted Eating: A Qualitative Study’. [file NDI-82-423-s001.docx]

Supplementary Materials

***Navigating Challenges and Adherence in Time-Restricted Eating:***

***A Qualitative Study***

Contents

- Page 2; Supplementary Table S1: Complete Guiding Questions
- Page 3; Supplementary Table S2: COREQ checklist

**Supplementary Table S1: Complete interview guiding questions**

| **Topic** | **Guiding Questions** |
| --- | --- |
| General Experience (in Weight Control) | **Before we talk about TRE, would you mind telling me about your previous experiences in controlling your weight?**  *Probing questions (only as needed)*   - *What methods have you tried in the past to control your weight? And what was your experience?* - *What were your considerations in choosing a particular method to control your weight?* |
| Reasons to Use Daily TRE | **Can you tell me what made you first decide to try TRE?**  *Probing questions (only as needed)*   - *Can you tell me your reasons for choosing TRE? What convinced you to start trying TRE?* - *Could you tell me were there any things that held you back from trying TRE?* |
| Personal Experience with TRE (Barriers and Facilitators) | **Can you describe a typical day for you when doing TRE, especially related to your meal timing?**  *Probing questions (only as needed)*   - *Is it always the same between days, or there is difference, for example weekdays and weekend?* - *What do you usually eat/drink in your eating window? and fasting window?*   **Can you tell me about your experiences in doing daily TRE so far?**  *Probing questions (only as needed)*   - *What are the advantages of TRE in your opinion compared to other methods you have tried?* - *What changes have you noticed in your health after doing TRE?* - *What are the impacts of TRE on your other health behaviors? (Eating pattern, physical activity, stress, sleep, etc.)* - *What impact have you experienced on your social life after doing TRE? How does it fit with your culture? (Compatibility with activities like Christmas, religious fasting, dinner)* - *What do you think makes you stick with TRE (eg. did you read scientific literature about TRE)?* - *Have you recommended TRE to others? If so, can you share the experience?*   **What are the obstacles/challenges that you experienced in doing TRE? How did you handle them?**   - *[if any inconsistent eating window] How do you feel when you do not stick to your TRE plan (e.g did you feel guilty)?* - *As the time went by, what changes have you felt in doing TRE?* |
| Family and Friends Experience with TRE (Barriers and Facilitators) | **What is your family or friends views about doing the TRE and how does it impact them**  *Probing questions (only as needed)*   - *Do they support you or push back?* - *What did others think of you doing TRE? Did you get any positive or negative comments?* - *How important is support to you and how do you find them?* |
| Future Plans | **What are your future plans to control your weight (and health)?**  *Probing questions (only as needed)*   - *How you would describe your plans with your dietary pattern, especially using TRE for the long term?* - *Will you continue doing TRE?* - *How confident are you in maintaining your weight loss going forward?* |
| Other | **Do you have anything else you would like to add about your weight and TRE experience?** |

**Supplementary Table S2. COREQ Checklist for manuscript “Navigating Challenges and Adherence in Time-Restricted Eating: A Qualitative Study”**

| **No** | **Item** | **Guide questions/description** | **Response** |
| --- | --- | --- | --- |
| **Domain 1: Research team and reflexivity** | | |  |
| Personal Characteristics | | |  |
| 1. | Interviewer/facilitator | Which author/s conducted the interview or focus group? | Mentioned in page 4 |
| 2. | Credentials | What were the researcher's credentials? *E.g. PhD, MD* | MD & PhD  mentioned in page 4 |
| 3. | Occupation | What was their occupation at the time of the study? | Mentioned in page 4 |
| 4. | Gender | Was the researcher male or female? | Mentioned in page 4 |
| 5. | Experience and training | What experience or training did the researcher have? | Mentioned in page 4 |
| Relationship with participants | | |  |
| 6. | Relationship established | Was a relationship established prior to study commencement? | No prior relationship was established with the participants before the study, Mentioned in page 4 |
| 7. | Participant knowledge of the interviewer | What did the participants know about the researcher? e*.g. personal goals, reasons for doing the research* | Explanation and participant information form was given to each participant before interview. |
| 8. | Interviewer characteristics | What characteristics were reported about the interviewer/facilitator? e.g. *Bias, assumptions, reasons and interests in the research topic* | Explanation and participant information form was given to each participant before interview, including interviewe interests in research topic |
| **Domain 2: study design** | | |  |
| Theoretical framework | | |  |
| 9. | Methodological orientation and Theory | What methodological orientation was stated to underpin the study? *e.g. grounded theory, discourse analysis, ethnography, phenomenology, content analysis* | *Content analysis*  Mentioned in methods section |
| Participant selection | | |  |
| 10. | Sampling | How were participants selected? *e.g. purposive, convenience, consecutive, snowball* | *Purposive*  Mentioned in the methods section |
| 11. | Method of approach | How were participants approached? e*.g. face-to-face, telephone, mail, email* | *Online, flyers, and radio interview*  Mentioned in the methods section |
| 12. | Sample size | How many participants were in the study? | 21  Mentioned in the method section and result |
| 13. | Non-participation | How many people refused to participate or dropped out? Reasons? | 0 |
| Setting | | |  |
| 14. | Setting of data collection | Where was the data collected? e*.g. home, clinic, workplace* | *Online and in university premises*  Mentioned in the method section |
| 15. | Presence of non-participants | Was anyone else present besides the participants and researchers? | No |
| 16. | Description of sample | What are the important characteristics of the sample? *e.g. demographic data, date* | Presented in Table 1 and narrative |
| Data collection | | |  |
| 17. | Interview guide | Were questions, prompts, guides provided by the authors? Was it pilot tested? | Yes, explained in methods section |
| 18. | Repeat interviews | Were repeat interviews carried out? If yes, how many? | No |
| 19. | Audio/visual recording | Did the research use audio or visual recording to collect the data? | Yes. Mentioned in the methods section |
| 20. | Field notes | Were field notes made during and/or after the interview or focus group? | Yes |
| 21. | Duration | What was the duration of the interviews or focus group? | 30-90 minutes, mentioned in the methods section |
| 22. | Data saturation | Was data saturation discussed? | Yes, mentioned in the method section |
| 23. | Transcripts returned | Were transcripts returned to participants for comment and/or correction? | Yes, mentioned in the method section |
| **Domain 3: analysis and findings** | | | |
| Data analysis | | | |
| 24. | Number of data coders | How many data coders coded the data? | Two data coders, mentioned in the methods section |
| 25. | Description of the coding tree | Did authors provide a description of the coding tree? | Description of coding process included in method section (page 4-5) |
| 26. | Derivation of themes | Were themes identified in advance or derived from the data? | Derived from the data, mentioned in the methods and discussion |
| 27. | Software | What software, if applicable, was used to manage the data? | NVivo 12, mentioned in the methods section |
| 28. | Participant checking | Did participants provide feedback on the findings? | Yes, member checking was performed |
| **Reporting** | | | |
| 29. | Quotations presented | Were participant quotations presented to illustrate the themes / findings? Was each quotation identified? e*.g. participant number* | Yes |
| 30. | Data and findings consistent | Was there consistency between the data presented and the findings? | Yes |
| 31. | Clarity of major themes | Were major themes clearly presented in the findings? | Yes |
| 32. | Clarity of minor themes | Is there a description of diverse cases or discussion of minor themes? | Yes, described in each themes. |
